# Towards hope

**DOI:** 10.1080/17571472.2017.1415188

**Published:** 2017-12-20

**Authors:** Chris Manning

**Affiliations:** Retired GP in West London; Convenor of the Action for NHS Workforce Wellbeing Network

You will all be familiar with the things that lift your spirits and those that drain them. I know what lifts mine and that is people who, in the face of the greatest odds hearken to and practice the words of Gandhi:

If we could change ourselves, the tendencies in the world would also change. As a man changes his own nature, so does the attitude of the world change towards him … . We need not wait to see what others do.^1^

And with Ghandi’s words in mind I present you with the ‘Why it matters to me’ statement that fronts up the Gupta et al. paper in this issue of London Journal of Primary Care. It is  a Statement of Progression and Declaration of Intent:

London Journal of Primary Care (LJPC) publishes articles on the multi-dimensional aspects of primary care that make it so human and vibrant. LJPC has grown a network of people who want to develop holistic, community-oriented integrated care and health promotion as a force for whole-society health.

LJPC is not so much a journal I would say. It is more like a movement that gives hope by championing and supporting a broad vision for health and care. It lifts our eyes beyond the single picture frame to view a whole gallery. It also provides opportunities to think freely and design systems and models of care that derive from the range of people’s needs and meet them, rather than simply describing and assessing focused aspects.

Further, LJPC works with many different people’s views and experiences and, having valued them, comes up with comprehensive solutions; the words on its lips being ‘collaboration’ and ‘co-production’. As Gupta et al. enunciate in this issue: ‘Primary care can have an enhanced effect on the good mental health of the population by collaborating with others within local communities for health’. It is affirming to know that such collaboration also benefits the efficiency and health of those doing the collaborating [1]. The enhanced effects of collaborative working on individual practice reminds us that Governments and leaders of all kinds often devise strategy that does the opposite, exemplifying individual actions that can isolate and demotivate others. As John Donne famously wrote in 1624, ‘No man is an island entire of itself’.^2^ We are all interconnected, even if dividable, and policy should help to achieve strong, healthy inter-connections between people for the sake of the whole of society.

Gupta et al. continue: ‘we hope that this paper will help general practice and more broadly, primary care, to take a strong role in developing this (force for whole-society health), in collaboration with public health, local authorities, healthcare and other organisations’. This aligns the paper to the increasing number of initiatives that enable collaboration, such as that being undertaken by The Health Foundation in its (UK Four Nation) Healthy Lives Strategy.^3^^,^^4^

Strategies are nothing without enactment. It is change that matters, beyond the words. LJPC has an important role to play here in supporting those who are committed to making a real difference in their own backyard. In the case of a GP, that means making a real difference to the person in front of them – one of the main duties and rewards of general practice

McCartney highlights in this Issue of LJPC, general practices can fail in their duties when we fail to see patients in their full humanity and complexity and instead simply apply guidelines from medical science:

By shifting our focus away from the computer, and back onto the person, talking about risks and harms, sharing uncertainties, and talking about priorities – the reason that GPs like me consider that general practice is the best job in the world will surely re-emerge.

McCartney’s hope is that

More honest medicine will result in less but higher value medicine … stopping doing things that don’t work, or work rarely, or come with an unacceptable burden of side effects or appointments should make room for the pleasure of practicing medicine.

McCartney continues: ‘Plato argued that practicing medicine according to a rulebook was second class medicine (for application to slaves). In today’s practice not following a guideline can seem like acting against a responsible body of medical opinion’. We must chart a course for general practice that makes sure that ‘evidence’ considers the breadth of issues that affect someone’s health, and not morph into protocols that encourage us to cover our backs and tick-off the ‘must-do’s’. It would be paradoxical indeed, at a time when ‘mindfulness’ is the word on so many people’s lips, that general practice procedures should encourage mindlessness.

Since we are ‘all people, first and foremost’ and ‘have a single condition that we all share – the human one’ (Seager. M – personal communication), it has always interested, and increasingly concerns me, that many doctors still seem to be trained out of their own humanity and emotions, or at least not helped to be aware of the fact that they are ‘wired’ the same and behave according to the laws of nature and psychodynamics as the people for whom they will be professionally caring. Segal wants to contribute to ‘understanding how people feel and react in the context of illness or damage to the body … not simply to understand the world but to change it’. In his Review of her book, Thomas states:

Even without illness, it seems to me that large numbers of adults (and pre-adults) exhibit the accusing features of the paranoid-schizoid position and the withdrawn features of depression. As a consequence they are poor at developing loving relationships. If they are doing this because of inner anxieties, the implications for care and prevention are far-reaching. They need to acknowledge that they are causing the harm from their personal phantasies and get help to change them. Everyone in society needs to know how to manage anxiety in healthy ways.

Mtandabari has some healing balm for us all in her paper.

Sometimes people know that they wish to make a change. However, they are simply not emotionally or practically ready or able to take change forward. This might be due to depression, anxiety or burnout. If you think this might apply to you, show yourself the same empathy you would show a patient. Feeling better is possible and achievable, but it can feel so challenging to take the first steps. Be kind to yourself and seek help either with your GP or one of the agencies below. Tell a loved one. Reach out to a friend.

Here, she reminds us that being healthy and finding hope involves doing things that are uncomfortable, things that may frighten us. The route to health means finding the courage to rise above adversity to reach another realm – ‘feel the fear and do it anyway’ to quote Susan Jeffers.

In the final paper of this Issue of LJPC, Corelli informs us that the Japanese artist Hokusai’s ‘extraordinary last painted works show that the artist had indeed reached a sublime realm in his beliefs and art’. Well, when did *you* last reach a ‘sublime realm’?

This old truth that good comes from overcoming ones fears and keeping a broad, optimistic view has recently been given new impetus by a paper published in the Journal of Consulting and Clinical Psychology. This paper includes the findings of one of the most extensive studies ever carried out in depression that compares psychological and drug/placebo treatments – the Treatment of Depression Collaborative Research Programme [2].

Expectancy of recovery is *the* key factor in recovering from depression. This finding is immensely important for everything we do in general practice. For one thing it drives engagement with the therapist – an outcome that was rediscovered in the IMPACT study by Goodyer et al. [3] – and for another, it is professionally and personally rewarding to have the required skills that can move people from despair towards hope and a life lived more healthily and well. After all, the original derivation of ‘wealth’ was from the words ‘well’ and ‘health’.^5^

The future of LJPC will be to develop collaborating sites that use their established communication lines and social media to engage more and more people in the process of co-creating hope and co-producing comprehensive solutions to complex human problems. Individuals within these collaborating sites need to be skilled at using approaches that dissolve fear and encourage healthy, collaborative living. Join in.

## Notes

1. 1964, The Collected Works of Mahatma Gandhi, Volume XII, April 1913 to December 1914, Chapter: General Knowledge About Health XXXII: Accidents Snake-Bite, (From Gujarati, Indian Opinion, 9-8-1913), Start Page 156, Quote Page 158, The Publications Division, Ministry of Information and Broadcasting, Government of India. (Collected Works of Mahatma Gandhi at gandhiheritageportal.org).

2. https://en.wikiquote.org/wiki/John_Donne (Accessed November 2017).

3. http://www.health.org.uk/publication/healthy-lives-people-uk (Accessed November 2017).

4. http://www.health.org.uk/sites/health/files/HealthyLivesForPeopleInTheUK.pdf (Accessed November 2017).

5. https://en.wikipedia.org/wiki/Wealth (Accessed November 2017).
